# Behavioral architecture of opioid reward and aversion in C57BL/6 substrains

**DOI:** 10.3389/fnbeh.2014.00450

**Published:** 2015-01-12

**Authors:** Stacey L. Kirkpatrick, Camron D. Bryant

**Affiliations:** Laboratory of Addiction Genetics, Pharmacology and Experimental Therapeutics, Boston University School of MedicineBoston, MA, USA

**Keywords:** factor analysis, motivated behavior, opioid, euphoria, dysphoria, naltrexone, substrain, quantitative trait locus

## Abstract

Drug liking vs. drug disliking is a subjective motivational measure in humans that assesses the addiction liability of drugs. Variation in this trait is hypothesized to influence vulnerability vs. resilience toward substance abuse disorders and likely contains a genetic component. In rodents and humans, conditioned place preference (CPP)/aversion (CPA) is a Pavlovian conditioning paradigm whereby a learned preference for the drug-paired environment is used to infer drug liking whereas a learned avoidance or aversion is used to infer drug disliking. C57BL/6 inbred mouse substrains are nearly genetically identical, yet demonstrate robust differences in addiction-relevant behaviors, including locomotor sensitization to cocaine and consumption of ethanol. Here, we tested the hypothesis that B6 substrains would demonstrate differences in the rewarding properties of the mu opioid receptor agonist oxycodone (5 mg/kg, i.p.) and the aversive properties of the opioid receptor antagonist naloxone (4 mg/kg, i.p.). Both substrains showed similar degrees of oxycodone-induced CPP; however, there was a three-fold enhancement of naloxone-induced CPA in agonist-naïve C57BL/6J relative to C57Bl/6NJ mice. Exploratory factor analysis of CPP and CPA identified unique factors that explain variance in behavioral expression of reward vs. aversion. “Conditioned Opioid-Like Behavior” was a reward-based factor whereby drug-free locomotor variables resembling opioid treatment co-varied with the degree of CPP. “Avoidance and Freezing” was an aversion-based factor, whereby the increase in the number of freezing bouts co-varied with the degree of aversion. These results provide new insight into the behavioral architecture of the motivational properties of opioids. Future studies will use quantitative trait locus mapping in B6 substrains to identify novel genetic factors that contribute to the marked strain difference in NAL-CPA.

## Introduction

Opioids, including the prescription mu opioid receptor agonist oxycodone (OXY) are powerfully addictive substances. The non-medical abuse of OXY fueled the two-fold increase in heroin use and dependence between 2007 and 2011 (http://www.samhsa.gov/) and opioid abuse has now reached epidemic proportions (Atluri et al., 2014; Mars et al., 2014). The addictions are heritable psychiatric diseases (Goldman et al., 2005; Ho et al., 2010; Ducci and Goldman, 2012); however, the major genetic factors have yet to be identified. Substance use disorders are defined by an aggregate of symptoms that can vary across time in their presence and severity. Many of these traits are likely to have different genetic architectures, making it difficult to identify the underlying causal factors of the addicted state.

The degree of subjective drug “liking” that individuals experience following drug administration is an intuitively simple phenotype that could influence susceptibility toward opioid addiction (Haertzen et al., 1983) and has been used to infer the addiction liability of opioids, including OXY formulations (Setnik et al., 2011; Webster et al., 2012). There is substantial variation among individuals with regard to opioid liking, with some individuals even reporting a clear disliking; interestingly, opioid disliking appears to have a stronger genetic component (Angst et al., 2012). An overarching hypothesis is that genetic factors responsible for variation in opioid liking vs. disliking contribute to individual differences in opioid addiction liability.

In model organisms and humans, drug reward can be assessed using a Pavlovian conditioning procedure known as conditioned place preference/conditioned place aversion (CPP/CPA) (Tzschentke, 2007; Childs and de Wit, 2009, 2013). Here, distinct environmental cues are explicitly paired with a context where either drug or saline (SAL) is administered and following repeated intermittent trials, subjects are subsequently given a choice between preference for or avoidance of the drug or SAL-paired environment. CPP for the drug-paired environment is predicted by self-reported measures of drug liking in humans (Childs and de Wit, 2009) and is used to infer the addiction liability of opioids (Hunter and Reid, 1983). CPA for the drug-paired environment is used to infer the dysphoric properties of drugs, including opioid receptor antagonists (Martin del Campo et al., 1992).

In contrast to mu opioid receptor agonists such as OXY that produce CPP (Bryant et al., 2012a), opioid receptor antagonists such as naloxone (NAL) produce a conditioned place aversion for the drug-paired environment (Mucha and Iversen, 1984; Mucha et al., 1985; Burgdorf et al., 2001; Sakoori and Murphy, 2004, 2005; Shoblock and Maidment, 2006). NAL-CPA requires mu opioid receptors (Skoubis et al., 2001) and endogenous enkephalins (Skoubis et al., 2005) but does not require D1 or D2 dopamine receptors (Narayanan et al., 2004). Sensitivity to NAL-CPA is greatly enhanced by prior chronic administration of mu opioid receptor agonists (Shoblock and Maidment, 2006) and represents a model for the negative affective-motivational component of opioid withdrawal (Schulteis et al., 1994). Furthermore, NAL and the closely related compound naltrexone cause dysphoria in a subset of human subjects, including healthy human volunteers (Martin del Campo et al., 1992) and opioid addicts (Crowley et al., 1985; Malcolm et al., 1987; Handelsman et al., 1992; Kanof et al., 1992). Finally, opioid receptor antagonists are used to treat craving in a subset of alcoholic patients (Oslin et al., 2006; Ray et al., 2010). Thus, an improved understanding of the genetic and neurobehavioral basis of both opioid reward and aversion has therapeutic relevance for prevention and treatment strategies in opioid and alcohol dependence.

Here, we present a detailed analysis of OXY-CPP and NAL-CPA in closely related C57BL/6 mouse substrains. C57BL/6J and C57BL/6NJ are nearly genetically identical (Keane et al., 2011), yet show robust behavioral differences in behavioral phenotypes relevant to psychiatric disorders (Bryant et al., 2008), which greatly facilitates the ability to map the underlying genetic factors (Bryant, 2011; Kumar et al., 2013). We examined standard CPP/CPA behavioral measures such as the change in time spent on the drug-paired side (Bryant et al., 2012a) in the context of other motivationally relevant behaviors, including the change in distance, number of visits, mean visit time, and freezing bouts on the side of preference (OXY) vs. the side of retreat (NAL) during the drug-free and drug-induced (state-dependent) OXY-CPP and NAL-CPA, respectively. Exploratory factor analysis was used for each strain following the induction of OXY-CPP vs. NAL-CPA in an effort to reduce a large number of variables into a smaller set of common factors. Here, we wished to gain new insight into the behavioral architecture that differentiates reward from aversion and to identify potential genetic differences.

## Materials and methods

### Mice

All experiments were performed in accordance with the National Institutes of Health Guidelines for the Use of Laboratory Animals and were approved by the Institutional Animal Care and Use Committee at Boston University. Female and male C57BL/6J and C57BL/6NJ mice were ordered from the Jackson Laboratory (Bar Harbor, ME USA) and were 8 weeks old at the beginning of behavioral testing. Upon arrival, mice were housed 2–4 per cage in standard shoebox-sized cages and were acclimated for 1 week to the vivarium. Mice were tested during the light phase of the light/dark cycle between 0800 and 1600 h. A 12 h/12 h light/dark cycle (lights off at 1830) was used for animal housing in the vivarium. Sample sizes for each treatment, strain, and sex are listed in Tables 1, **3**.

**Table 1:** **Change in behavior on the OXY-paired preference side (right side) following SAL or OXY training**.

**SAL training**		
**Variable**	**J (S.E.M.) *N* = *56* (30 F, 26 M)**	**NJ (S.E.M.) *N* = *45* (25 F, 20 M)**
D8-D1 time (s)	17.6 (21.5)	12.1 (23.0)
D8-D1 visit time (s)	1.1 (1.1)	3.5 (3.3)
D8-D1 visits	−4.7 (3.5)	−0.3 (2.9)
D8-D1 rotations	−7.2 (1.3)	−4.2 (1.6)
D8-D1 distance (m)	−3.9 (1.1)	−2.8 (1.1)
D8-D1 freezing bouts	9.8 (16.4)	−8.5 (17.0)
D9-D1 time (s)	−0.7 (28.0)	−3.6 (29.9)
D9-D1 visit time (s)	2.1 (1.0)	1.9 (1.0)
D9-D1 visits	−11.4 (4.0)	−5.9 (2.6)
D9-D1 rotations	−6.8 (2.3)	−6.6 (1.7)
D9-D1 distance (m)	−6.2 (1.8)	−5.6 (1.1)
D9-D1 freezing bouts	7.2 (17.3)	6.4 (15.1)
**OXY training**		
**Variable**	**J (S.E.M.) *N* = *59* (29 F, 30 M)**	**NJ (S.E.M.) *N* = *55* (28 F, 27 M)**
D8-D1 time (s; CPP)	107.9 (29.9)	105.0 (25.0)
D8-D1 visit time (s)	3.4 (0.9)	1.5 (0.6)
D8-D1 visits	−8.1 (2.1)	1.0 (2.7)
D8-D1 rotations	−2.2 (1.3)	0.6 (1.4)
D8-D1 distance (m)	−1.3 (1.0)	1.2 (0.9)
D8-D1 freezing bouts	35.5 (16.4)	7.7 (18.5)
D9-D1 time (s)	238.7 (34.6)	291.5 (42.9)
D9-D1 visit time (s)	−3.2 (3.0)	31.3 (32.6)
D9-D1 visits	161.6 (13.6)	148.3 (17.0)
D9-D1 rotations	189.9 (13.8)	198.7 (15.2)
D9-D1 distance (m)	117.7 (5.9)	116.2 (6.9)
D9-D1 freezing bouts	−56.4 (13.7)	−45.5 (19.8)

*The mean ± S.E.M. and sample size (N; F, females; M, males) for each strain (J, NJ) and treatment (SAL, OXY) is presented for each of the 12 variables used in factor analysis*.

### Drugs

OXY was purchased from Sigma-Aldrich (St. Louis, MO) and NAL was purchased from Tocris Bioscience (Bristol, UK). The dose of OXY (5 mg/kg, i.p.) was chosen based on our previous study (Bryant et al., 2012a) and based on several other studies employing similar doses (3–10 mg/kg) to induce a reliable CPP (Liu et al., 2009; Rutten et al., 2010, 2011a,b; Niikura et al., 2013). Furthermore, pilot data from both strains indicated a more pronounced CPP and locomotor stimulatory effect of 5 mg/kg OXY relative to lower doses (data not shown). The dose of NAL (4 mg/kg, i.p.) was chosen based on previous studies employing a dose range of 4–10 mg/kg (Skoubis et al., 2001, 2005; Narayanan et al., 2004; Sakoori and Murphy, 2004, 2005, 2008; Solecki et al., 2009) and based on pilot studies indicating that this dose was more effective at inducing CPA than 2 mg/kg (data not shown).

### CPP/CPA

**Figure 1:**
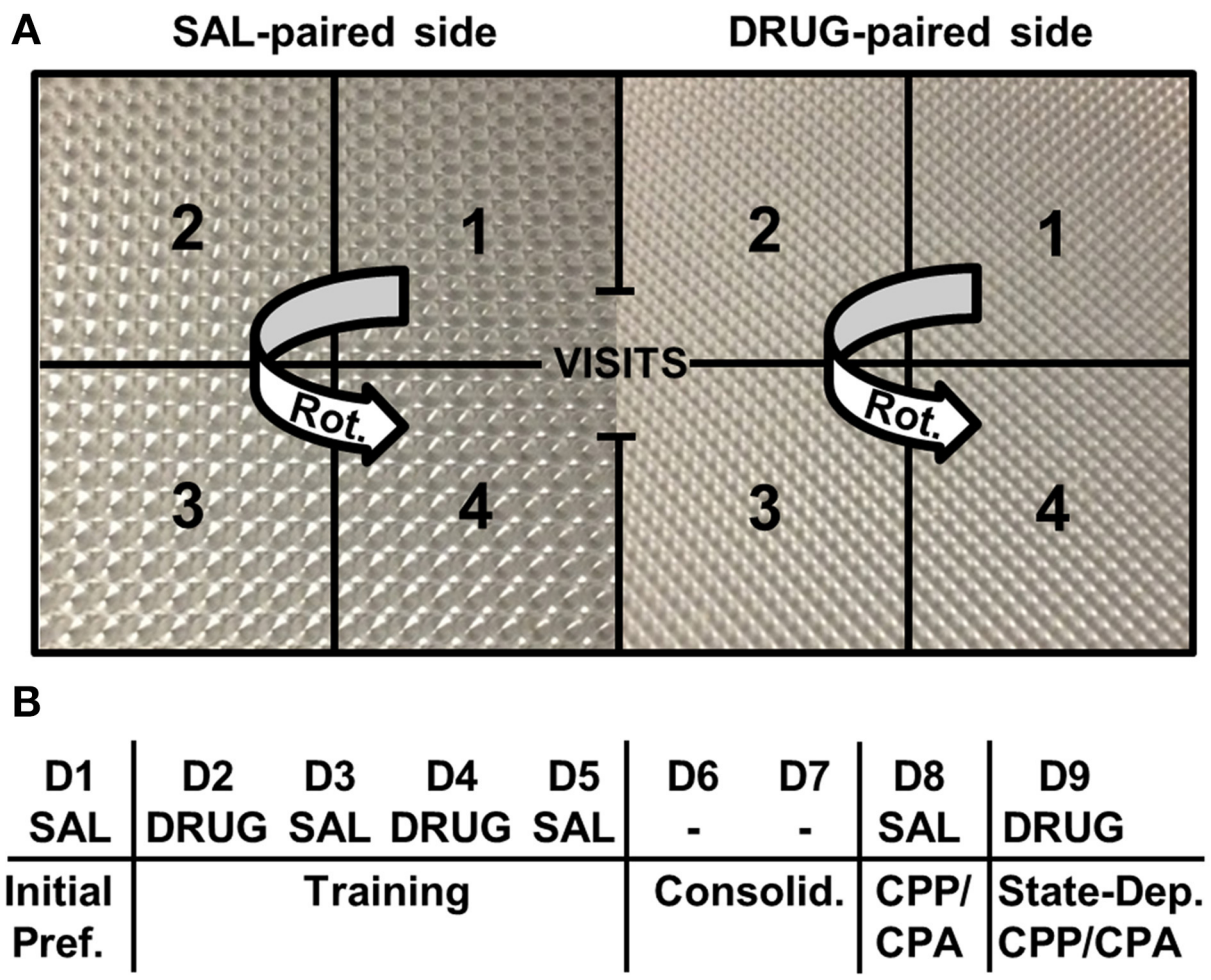
**Place conditioning chambers and CPP/CPA design. (A)** A two-chamber design was employed whereby following initial assessment of preference for each side, drug injections were paired with the right side and saline injections were paired with the left side. Different floor textures were used as contextual cues. Notably, the number of rotations (Rot.) was measured on each side. A rotation was defined by the completion of a unidirectional, circular sequence across the four zones starting from any zone number and rotating in either direction (e.g., 1-2-3-4 or 3-2-1-4). **(B)** CPP/ CPA protocol. Initial Pref., initial preference for the drug-paired side; Consolid., consolidation period whereby mice were left undisturbed in their home cages in the vivarium for 2 days; State-Dep., state-dependent assessment of OXY-CPP or NAL-CPA whereby mice were administered a subsequent drug injection and tested for CPP/CPA; D, day of training; SAL, saline; DRUG, OXY or NAL treatment; -, no treatment was administered.

We used an unbiased, two-chamber design (Bryant et al., 2012a,b,c). Mice were recorded in unlit CPP chambers using infrared cameras (Swann Communications U.S.A., Inc., Sante Fe Springs, CA, USA) that were mounted to the ceiling of sound attenuating chambers (Med Associates, St. Albans, VT). Each Plexiglas side of the chamber was 20 cm in length by 20 cm in width by 46 cm in height whereby the two sides of the conditioning apparatus (separated by an ion transparent black divider) differed only in the type of floor texture of the plastic inserts (Plaskolite, Inc. Columbus, OH) (Figure 1A). Mice were habituated for a minimum of 1 h in the testing room prior to starting the experiment. On Day 1, all mice were assessed for initial preference for the right-paired side (the eventual drug-paired side) whereby mice received SAL (i.p.), were placed into the left side (the eventual SAL-paired side), and were provided free access to both sides for 30 min. On Days 2 and 4, mice received an injection of either SAL (i.p.), OXY (5 mg/kg, i.p.), or NAL (4 mg/kg, i.p.) and were confined to the right side for 30 min. On Days 3 and 5, all mice received SAL (i.p.) and were confined to the left side for 30 min. Days 6 and 7 served as a 2-day consolidation period whereby mice were left undisturbed in their home cage in the vivarium. On Day 8, mice were tested for drug-free CPP/CPA whereby all mice received SAL (i.p.), were placed into the left side, and were provided free access to both sides for 30 min (Figure 1B). On Day 9 mice were tested for state-dependent CPP/CPA (Mucha and Iversen, 1984) whereby mice were injected with the same treatment they received during drug training on Days 2 and 4 (SAL, OXY, or NAL; Figure 1B). Behavioral data were tracked using Anymaze video tracking software (Stoelting Co., Wood Dale, IL). Our primary outcome variables for the study included the change in time spent on the drug-paired side between Days 1 and 8 (D8-D1; drug-free CPP/CPA) and between Days 1 and 9 (D9-D1; state-dependent CPP/CPA). Additional variables were measured as described below. Data were exported to an Excel spreadsheet, curated into a dataframe, and statistically analyzed in R (http://www.r-project.org/).

### Behavioral analysis

We examined potential strain differences in drug-induced responses between drug training Days 2 and 4 (D4-D2; Figure 1B). In considering D4-D2 distance, rotations (Figure 1A), and freezing bouts, we ran Three-Way ANOVAs (Treatment, Strain, Sex). There were always two levels of treatment; either OXY vs. SAL or NAL vs. SAL. Two-way interactions (Treatment, Strain) were further investigated with *t*-tests using significance thresholds that were Bonferroni-corrected for the number of specified comparisons (see below).

With regard to the subtraction variables for OXY vs. SAL, we wanted to know the behavioral structure of the expression of opioid reward; thus, we focused on the OXY (right)-paired side—the preference side. With regard to NAL vs. SAL, we wanted to know the behavioral structure comprising opioid avoidance / aversion; thus for this analysis, we turned our focus toward the SAL-paired side which we refer to as the side of retreat. Our rationale behind using the SAL-paired side as the point of reference for NAL-induced conditioned aversive behaviors is that in this case, the non-drug side is where the mice spent a majority of their time “behaving” during CPA assessment and presumably where the motivational expression of aversion/dysphoria occurs. Thus, we felt it was most appropriate to examine the behavior in the specific environment where the mice are expressing the avoidance of NAL, or i.e., the side of retreat (just as we are examining the specific environment where the mice are expressing preference for OXY). Nevertheless, behaviors and factor analyses of NAL-trained mice on the NAL side can be found in Supplementary Tables S1, S2. Three-Way ANOVAs (Treatment, Strain, and Sex) were conducted and we only observed one significant three-way interaction with regard to OXY vs. SAL (D9-D1 visits; data not shown). We did not observe any three-way interactions with regard to NAL vs. SAL. Therefore, for all subtraction variables, we collapsed across Sex and focused on two-way interactions followed by *t*-tests whereby the significance thresholds were adjusted for the four main comparisons (*p* < 0.0125; 0.05/4 = 0.0125). The four comparisons included (1) the strain comparison in SAL-trained mice; (2) the strain comparison in drug-treated mice; (3) the treatment comparison for the J strain; and (4) the treatment comparison for the NJ strain.

### Exploratory factor analysis

Exploratory factor analysis is a multivariate statistical method used to reduce the data from a larger set of variables into smaller sets of intercorrelated variables called common factors, providing insight into the underlying data structure. Video tracking analysis permits the ability to generate nearly an infinite number of variables containing varying degrees of correlation that can contribute to our understanding of the primary outcome variables under consideration: D8-D1 time and D9-D1 time. We included 12 variables in each factor analysis, including six main variables [distance, time, mean visit time, visits, rotations (see description for rotations in Figure 1A)], and freezing bouts (defined as a minimum of 250 ms of immobility) that comprised six subtraction variables during drug-free CPP/CPA assessment (D8-D1) and state-dependent CPP/CPA assessment (D9-D1). Factor analysis was conducted for OXY-trained vs. SAL-trained mice for the OXY (right)-paired side (the side where preference-related behaviors were expressed) and for NAL-trained vs. SAL-trained mice for the SAL (left)-paired side (the side of retreat where avoidance-related behaviors were expressed).

Exploratory factor analysis was conducted with the R package “psych” using the “fa” factor analysis function. Variables were standardized to z scores prior to generating the correlation matrix. We employed a minimum residual fitting procedure (“minres”) to minimize the squared residual of the factor model. We used the “Varimax” function for orthogonal rotation of the matrix whereby the solution for data reduction does not allow the factors to be correlated. Varimax rotation maximizes the sum of the variances of the squared correlations (loadings) between variables and factors, yielding a simple structure to the data that most efficiently loads each variable onto as few factors as possible. Factors with eigenvalues greater than one were included in the analysis. Using these criteria, the 12 behavioral variables (see below) loaded onto anywhere between three to five factors, depending on the treatment and/or strain.

## Results

### Behavioral analysis

F statistics from ANOVAs for treatment x strain interactions and main effects where relevant are reported followed by *t*-tests for the relevant comparisons. Means and S.E.M. are provided for the 12 subtraction variables for OXY vs. SAL in Table 1 (right side; preference side) and for NAL vs. SAL in **Table 3** (left side; the side of retreat).

#### Days 2 and 4 of OXY-CPP training

In comparing changes in behavior in OXY- vs. SAL-treated mice between Days 2 and 4 of training, we did not observe any three-way (Treatment, Strain, Sex) or two-way (Treatment, Strain) interactions as measured via changes in distance, rotations, or freezing bouts [*F*_(1, 207)_ < 1] nor did we observe any interactions when ANOVAs were run for the individual Day 2 or Day 4 variables [*F*_(1, 207)_ ≤ 1; data not shown].

#### OXY-CPP and state-dependent CPP

For both D8-D1 time and D9-D1 time, there was a main effect of Treatment [*F*_(1, 207)_ = 12.7, 54.9; *p* = 0.004, 3.6 × 10^−33^], indicating significant drug-free and state-dependent OXY-CPP (Table 1) that was not dependent on Strain [*F*_(1, 207)_ < 1] nor a Treatment × Strain interaction [*F*_(1, 207)_ < 1]. To summarize, we did not observe any strain differences in our primary outcome measures of opioid reward.

#### Days 2 and 4 of NAL-CPA training

**Figure 2:**
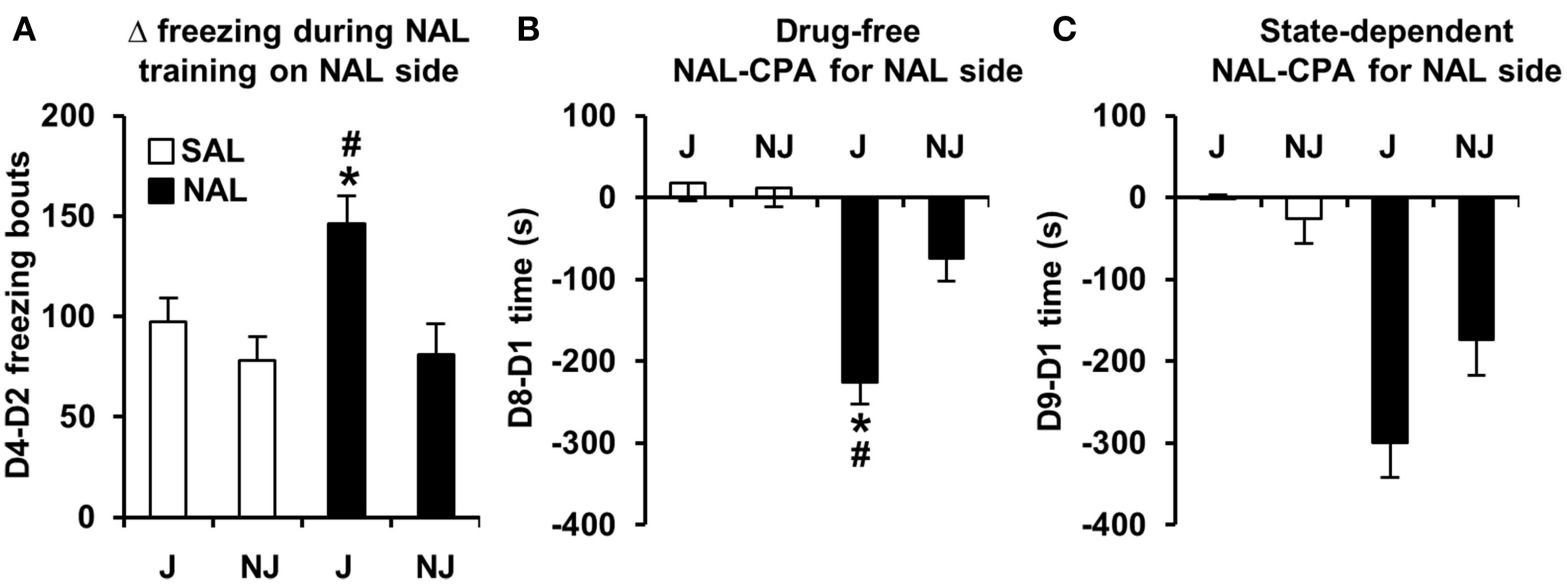
**Development of freezing bouts in J mice during NAL training predicts NAL-CPA for the NAL-paired side. (A)** NAL-trained J mice, but not NAL-trained NJ mice (black bars) demonstrated a significant instatement of NAL-induced freezing bouts from the first to second training trial relative to SAL-trained J mice (white bars). **(B,C)** This behavior predicted the strain-dependent enhancement of drug-free and state-dependent NAL-CPA. ^*^Significantly different from SAL control mice (*p* < 0.0125; Bonferroni-corrected significance threshold). ^#^Significantly different from NAL-trained NJ mice (*p* < 0.0125). Data are presented as the mean ± S.E.M.

In examining strain differences in behavioral training with NAL vs. SAL between Days 2 and 4, there was a significant Treatment x Strain interaction for D4-D2 freezing bouts [*F*_(1, 180)_ = 3.9; *p* = 0.05] that was explained by NAL-trained J mice showing an increase in the number of freezing bouts relative to SAL-trained J mice [*t*_(98)_ = 3.2; *p* = 0.0017] and relative to NAL-trained NJ mice [*t*_(85)_ = 3.1; *p* = 0.0023; Figure 2A]. In contrast, NAL-trained NJ mice did not show an increase in the number of freezing bouts relative to SAL-trained NJ mice [*t*_(87)_ < 1; Figure 2A]. Importantly, there was no strain difference between SAL-trained mice, indicating that this change in behavior was specific to a previous history of NAL treatment.

#### NAL-CPA and concomitant variables

**Figure 3:**
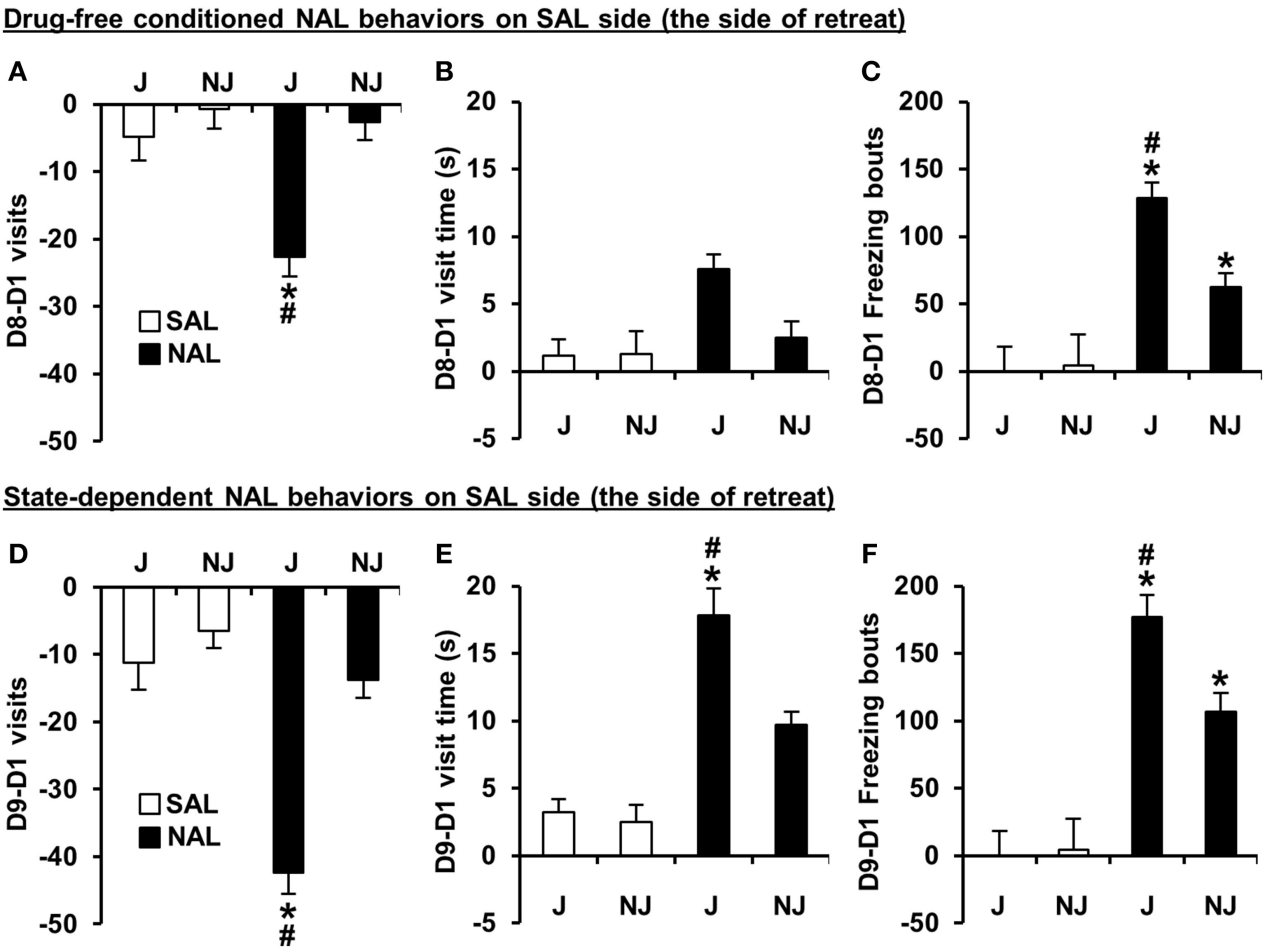
**Conditioned and state-dependent avoidance behaviors on the SAL-paired side (the side of retreat) during NAL-CPA. (A–C)** Conditioned, drug-free changes in visits, visit time, and freezing bouts on the SAL-paired side from Day 1 to Day 8 (D8-D1) in NAL-trained (black bars) vs. SAL-trained mice (white bars). **(D–F)** State-dependent changes in the number of visits, visit time, and freezing bouts on the SAL-paired side from Day 1 to Day 9 (D9-D1) following an injection of the treatment that was received during Days 2 and 4 of training [either NAL (4 mg/kg, i.p. black bars) or SAL (i.p. white bars)]. ^*^Significantly different from SAL control mice (*p* < 0.0125; Bonferroni-corrected significance threshold). ^#^Significantly different from NAL-trained NJ mice (*p* < 0.0125). Data are presented as the mean ± S.E.M.

For the SAL-paired side (the side of retreat), there was a significant Treatment × Strain interaction for D8-D1 time and D8-D1 visits [*F*_(1, 180)_ = 9.8, 7.6; *p* = 0.002, 0.006]. The D8-D1 time interaction was explained by NAL-trained J mice exhibiting a significant increase in time spent on the side of retreat (or i.e., a decrease in time spent on the NAL-paired side as illustrated in Figure 2B) relative to SAL-trained J mice [*t*_(98)_ = 7.1; *p* = 2.5 × 10^−10^] and relative to NAL-trained NJ mice [*t*_(85)_ = 3.9; *p* = 0.0002; Figure 2B]. NAL-trained NJ mice did not show significant NAL-CPA relative to SAL-trained NJ mice [*t*_(86)_ =2.2, *p* > 0.0125; Figure 2B]. The interaction for D8-D1 visits was explained by NAL-trained J mice exhibiting a significant decrease in visits relative to SAL-trained J mice [*t*_(98)_ = 4.8; *p* = 5.9 × 10^−6^] and relative to NAL-trained NJ mice [*t*_(85)_ = 5.2; *p* = 1.4 × 10^−6^; Figure 3A]. Importantly, there were no strain differences between SAL-trained mice for D8-D1 time or visits (Figures 2B, 3A), demonstrating the specificity of the strain difference to prior NAL training. Although the Treatment x Strain interaction was not significant for D8-D1 visit time [*F*_(1, 180)_ = 2.9; *p* = 0.092; Figure 3B], we present the results so that they can be compared with the state-dependent results in Figure 3E (see below).

For D8-D1 freezing bouts, the Treatment x Strain interaction was not significant [*F*_(1, 180)_ = 2.2; *p* = 0.14]; however, NAL-trained J mice clearly demonstrated a highly significant increase in the number of freezing bouts relative to SAL-trained J mice [*t*_(98)_ = 5.8; *p* = 1.1 × 10^−7^] and relative to NAL-trained NJ mice [*t*_(85)_ = 4.2; *p* = 7.0 × 10^−5^]. NAL-trained NJ mice also showed a significant increase in the number of freezing bouts relative to SAL-trained NJ mice [*t*_(86)_ = 3.2; *p* = 0.002; Figure 3C], contributing to the lack of Treatment × Strain interaction. Each pair-wise comparison survived the corrected significance threshold for multiple comparisons (*p* < 0.0125); thus, we conclude that J mice are more sensitive to NAL-induced conditioned freezing bouts that can be predicted by the J-specific development of freezing bouts during NAL training (Figure 2A).

#### State-dependent NAL-CPA and concomitant variables

For D9-D1 on the SAL-paired side, significant Treatment x Strain interactions were identified for D9-D1 visits, visit time, and freezing bouts [*F*_(1, 180)_ = 13.0, 5.2, 4.0; *p* = 0.004, 0.023, 0.046]. These interactions were explained by NAL-trained J mice showing a decrease in visits, an increase in visit time, and an increase in freezing bouts relative to SAL-trained J mice [*t*_(98)_ = 5.9, 6.3, 6.8; *p* = 6.3 × 10^−8^, 2.1 × 10^−8^, 1.1 × 10^−9^] and relative to NAL-trained NJ mice [*t*_(85)_ = 7.0, 2.8, 3.2; *p* = 6.4 × 10^−10^, 0.0055, 0.0017; Figures 3D–F]. NAL-trained NJ mice also showed a significant increase in freezing bouts relative to SAL-trained NJ mice [*t*_(86)_ = 3.4; *p* = 0.001; Figure 3F] but not any of the other behaviors (*p* > 0.0125; Figures 3D,E). To summarize, J mice are also more sensitive to the concomitant behaviors that support state-dependent NAL-CPA.

### Factor analysis

#### SAL-trained mice: Right and left sides

With regard to SAL-trained J mice, we identified four identical factors between the right side (OXY preference side) and left side (NAL side of retreat) that we named “Δ Time,” Δ Freezing,” “D8-D1 Activity,” and “D9-D1 Activity” (Tables 2A, **4A**). For NJ mice, the behavioral structure of the left side (side of retreat) was identical to the J strain (**Table 4B**) and contained the same four factors. However, for the right side (preference side), change in time loaded with additional variables, leading us to re-name this factor “Δ Time and Rotations” that also included D9-D1 distance (Table 2B). Accordingly, we also re-named the D9-D1 Activity factor to, “D9-D1 Visit Behavior” (Table 2B).

**Table 2:** **Factor analysis of OXY-CPP on OXY-paired preference side (right side)**.

**(A) SAL-trained B6J mice**						**(B) SAL-trained B6NJ mice**				
	**Δ Time**	**Δ Freez**.	**D8-D1 Act**.	**D9-D1 Act**.	**–**	**Δ Time and Rot**.	**Δ Freez**.	**D8-D1 Act**.	**D9-D1 Visit Beh**.	**–**
% variance	20.0	18.0	23.6	21.8		26.9	16.5	15.3	16.7	
D8-D1 time	0.74					0.52				
D8-D1 visit time			−0.65					−0.87		
D8-D1 visits			0.85					0.53		
D8-D1 rot.			0.64			0.52				
D8-D1 dist.			0.95					0.65		
D8-D1 freez.		0.98					0.99			
D9-D1 time	0.79					0.69				
D9-D1 visit time	0.83								0.99	
D9-D1 visits				0.77					−0.64	
D9-D1 rot.				0.91		0.65				
D9-D1 dist.				0.98		0.86				
D9-D1 freez.		0.93					0.91			
**(C) OXY-trained B6J mice**						**(D) OXY-trained B6NJ mice**					
	**Cond. Opioid-Like Beh**.	**State-Dep Pref**.	**State-Dep Opioid Act**.	**Δ Freez**.	**D8-D1 Visit Time**	**Cond. Opioid-Like Beh**.	**State-Dep Pref**.	**State-Dep Opioid Act**.	**Δ Freez**.	**D8-D1 Visit Beh**.
% variance	18.8	16.0	16.4	13.9	13.6	21.7	12.6	16.6	19.3	16.5
D8-D1 time	0.58					0.85				
D8-D1 visit time					0.97					0.91
D8-D1 visits	0.61									−0.90
D8-D1 rot.	0.69					0.84				
D8-D1 dist.	0.95					0.85				
D8-D1 freez.				0.98					0.81	
D9-D1 time		0.72					0.66			
D9-D1 visit time		0.58					0.84			
D9-D1 visits		−0.95							−0.64	
D9-D1 rot.			0.81					0.95		
D9-D1 dist.			0.96					0.92		
D9-D1 freez.				0.75					0.91	

*Freez., freezing bouts; Act., activity, Cond., conditioned; Dep., dependent; Beh., behavior; Pref., preference; dist., distance; rot., rotations*.

#### OXY-trained mice: OXY-paired side (preference side)

With regard to drug-free OXY-CPP (D8-D1 time), D8-D1 distance and D8-D1 rotations all loaded onto the same factor for both strains. We named this factor, “Conditioned Opioid-Like Behavior” (Tables 2C,D). Importantly, co-loading of D8-D1 time with opioid-like activity was specific to the OXY-paired side—for the SAL-paired side, there were distinct “D8-D1 Time” and “D8-D1 Activity” factors (data not shown). These observations indicate that conditioned reward and conditioned opioid-like activity measures co-vary in the drug-paired context. For J mice, D8-D1 visits also loaded positively onto “Conditioned Opioid-Like Behavior” (Table 2C). For NJ mice, D8-D1 visits loaded negatively with D8-D1 visit time onto a factor that we termed, “D8-D1 Visit Behavior” (Table 2D).

With regard to state-dependent OXY-CPP, D9-D1 time and D9-D1 visit time loaded onto a single factor for both J and NJ that we named, “State-Dependent Preference” (Tables 2C,D). Furthermore, for J mice, D9-D1 visits loaded negatively onto this same factor whereas for NJ mice, D9-D1 visits loaded onto Δ Freezing. For both strains, D9-D1 distance and rotations clearly loaded onto a single, discrete factor which we named, “State-Dependent Opioid Activity” (Tables 2C,D).

Similar to SAL-trained mice (Tables 2A,B, **4A,B**), for OXY-trained J and NJ mice, the change in freezing bouts for Days 8 and 9 loaded onto a “Δ Freezing” factor (Tables 2C,D), indicating that freezing is not associated with the expression of conditioned or state-dependent opioid reward.

#### NAL-trained mice: SAL-paired side (the side of retreat)

For aversion-prone J mice (Figure 2B), the relationship between aversion and freezing was extensive whereby a single factor contained conditioned (D8-D1) and state-dependent (D9-D1) changes in time and freezing bouts as well as visit time; we named this factor, “Avoidance and Freezing” (**Table 4C**). For the less aversive NJ strain (Figure 2B), “Conditioned Avoidance and Freezing” and “State-Dependent Avoidance and Freezing” formed separate factors (**Table 4D**). With regard to activity measures, for the aversion-prone J strain, there were clearly two distinct activity factors that we termed “D8-D1 Activity” and “D9-D1 Activity” (**Table 4C**) whereas for the less aversive NJ strain, there was a single “Δ Activity” factor for both days (**Table 4D**).

Behavioral values and factor analysis of NAL-trained mice on the NAL-paired side can be found in Supplementary Tables S1, S2. The results are similar whereby freezing loads onto measures of time for both strains (Table S2) rather than loading by itself in SAL-treated mice (Table 2).

## Discussion

B6 substrains showed a comparable level of opioid reward as measured via drug-free and state-dependent OXY-CPP (Table 1). However, J mice demonstrated a three-fold enhanced sensitivity to opioid aversion relative to NJ mice as measured via NAL-CPA (Table 3; Figure 2). The selectivity of the strain difference for the aversive properties of an opioid receptor antagonist and not the rewarding properties of a mu opioid receptor agonist suggests a difference in tonic release of endogenous opioids (Skoubis et al., 2005) or perhaps stress-induced release of endogenous opioids during CPP training that, in turn, leads to an adaptation at the receptor level [e.g., constitutive activity of the mu opioid receptor (Shoblock and Maidment, 2006)] or at the downstream level of the aversion neurocircuitry. In support, endogenous enkephalins are necessary for NAL-CPA (Skoubis et al., 2005) but not opioid-induced CPP (Marquez et al., 2006) whereas mu opioid receptors are necessary for both phenotypes (Matthes et al., 1996; Skoubis et al., 2001). Interestingly, NAL-CPA-prone J mice also drink more ethanol than C57BL/6N mice from the Charles River vendor (Bryant et al., 2008) and this behavior is in part mediated by endogenous opioids (Racz et al., 2008). Future studies will examine the potential for B6 strain differences in basal and drug-induced endogenous opioid levels as a potential neurochemical mechanism for variation in motivational behaviors.

**Table 3:** **Change in behavior on the SAL-paired side (left side; the side of retreat) following SAL or NAL training**.

**SAL training**		
**Variable**	**J (S.E.M.) *N* = *56* (30 F, 26 M)**	**NJ (S.E.M.) *N* = *45* (25 F, 20 M)**
D8-D1 time (s)	−17.6 (20.1)	−12.0 (23.0)
D8-D1 visit time (s)	1.2 (1.2)	1.3 (1.7)
D8-D1 visits	−4.8 (3.5)	−0.7 (2.9)
D8-D1 rotations	−7.0 (1.4)	−6.6 (1.6)
D8-D1 distance (m)	−6.6 (1.1)	−5.1 (1.1)
D8-D1 freezing bouts	0.1 (18.7)	−9.5 (22.0)
D9-D1 time (s)	−0.1 (28.0)	3.6 (29.9)
D9-D1 visit time (s)	3.2 (1.0)	2.5 (1.3)
D9-D1 visits	−11.3 (4.0)	−6.5 (2.6)
D9-D1 rotations	−5.5 (2.6)	−8.3 (1.7)
D9-D1 distance (m)	−8.1 (1.7)	−6.9 (1.0)
D9-D1 freezing bouts	−0.4 (19.0)	4.7 (22.6)
**NAL training**		
**Variable**	**J (S.E.M.) *N* = *44* (22 F, 22 M)**	**NJ (S.E.M.) *N* = *43* (23 F, 20 M)**
D8-D1 time (s)	225.5 (26.5)	73.8 (28.6)
D8-D1 visit time (s)	7.6 (1.1)	2.5 (1.2)
D8-D1 visits	−22.6 (2.9)	−2.7 (2.6)
D8-D1 rotations	−10.1 (1.7)	−5.9 (2.0)
D8-D1 distance (m)	−9.1 (1.0)	−5.8 (1.0)
D8-D1 freezing bouts	128.3 (11.9)	62.3 (10.4)
D9-D1 time (s)	299.6 (42.4)	174.2 (42.9)
D9-D1 visit time (s)	17.8 (2.0)	9.7 (1.0)
D9-D1 visits	−42.4 (3.1)	−13.8 (2.7)
D9-D1 rotations	−13.3 (1.9)	−9.1 (1.8)
D9-D1 distance (m)	−15.9 (1.2)	−9.8 (1.1)
D9-D1 freezing bouts	177.1 (16.5)	107.0 (14.0)

*The mean ± S.E.M. and sample size (N; F, females; M, males) for each strain (J, NJ) and treatment (SAL, NAL) is presented for each of the 12 variables used in factor analysis*.

Irrespective of strain, we observed very different behavioral architectures underlying OXY preference vs. NAL aversion. Following OXY training, OXY-CPP (D8-D1 time) loaded onto the same factor as change in distance and rotations for both strains (Tables 2A,B). Opioid-induced rotations (Figure 1A), or “circling” is a stereotypic behavior that is induced by opioid administration (Iwamoto and Way, 1977; Morihisa and Glick, 1977; Seidel et al., 1979; Mickley et al., 1990; Bryant et al., 2009; Hodgson et al., 2010) and its behavioral pattern can be expressed as a conditioned opioid-like placebo response (Bryant et al., 2009). Our observations indicate that mice expressing drug-free conditioned opioid reward behave in a manner that is similar to having received an opioid injection. This “Conditioned Opioid-Like Behavior” factor was context-specific; on the SAL-paired side this factor dissolved into two separate activity- and time-based factors (data not shown), similar to SAL control mice (Table 2). We hypothesize that expectation-induced placebo-like behavioral responding both contribute to and in turn, are reinforced by the conditioned, incentive-motivational properties of the tactile cues (Figure 1A) that were paired with OXY (Meyer et al., 2012), thus accounting for OXY-CPP.

With regard to state-dependent OXY-CPP following an OXY challenge (D9-D1 time), Conditioned Opioid-Like Behavior dissolved into separate factors that we termed State-Dependent Opioid Activity and State-Dependent Preference (Tables 2C,D). I.e., under the influence of OXY, opioid-induced activity was dissociable from conditioned reward. Interestingly, although there was a large, state-dependent increase in the number of visits following OXY administration that was accompanied by a two to three-fold enhancement of OXY-CPP in both strains (D9-D1 vs. D8-D1; Table 1), here, in J mice the change in visits loaded negatively (−0.95) onto the State-Dependent Preference factor containing time and visit time rather than positively (0.60) as was observed during drug-free OXY-CPP (Table 2C). We hypothesize that the positive link between visits and preference during the drug-free state (D8-D1) identifies “visits” as a drug seeking behavior in the absence of drug. On Day 9 (D9-D1), the need for drug seeking is obviated because mice are under the influence of OXY.

During NAL training for NAL-CPA, we discovered that although acute NAL treatment did not induce any change in the number of freezing bouts relative to SAL-treated mice on Day 2, following the second injection on Day 4, the J strain, but not the NJ strain showed a significant increase in the number of freezing bouts relative to the first NAL administration and relative to SAL-treated mice (Figure 2A). There are two possible explanations for this result. One is that there is a neuropharmacological instatement of NAL-induced freezing bouts that requires prior NAL exposure, resulting in a behavioral pharmacological response that is akin to a sensitization phenomenon. A second possibility is that this behavior represents a Pavlovian conditioned response that is expressed in anticipation of an aversive stimulus (naloxone)—similar to context-dependent freezing in anticipation of an aversive electric shock (Curzon et al., 2009). Given the present data, it is not possible to conclude whether the induction of freezing bouts during NAL training is a learned response because our experimental design did not contain a treatment group that received a NAL injection on Day 2 and a SAL injection on Day 4. Nevertheless, the subsequent results on Day 8 support the hypothesis that the development of a J-specific increase in freezing bouts during NAL training is not a neuropharmacological response, but rather, is a learned motivational response that is subsequently expressed to a greater degree in J mice vs. NJ mice in the absence of NAL (Figure 3C). Thus, in this case strain differences (and likely genetic differences) in the development of freezing bouts during behavioral training with NAL can be used to predict strain differences in behavioral expression of the dysphoric properties of NAL as measured via NAL-CPA (Figure 2B) and other concomitant behaviors (Figure 3).

We observed a robust strain difference in NAL-CPA (Figure 2B) and very different factor loadings for NAL-CPA vs. OXY-CPP (Tables 4C,D vs. Tables 2C,D). In the aversion-prone J strain, NAL-CPA during both the drug-free (D8-D1) and drug-induced state (D9-D1) loaded onto a single, distinct “Avoidance and Freezing” factor that contained an increase in time spent avoiding the NAL-paired side (Figures 2B,C; Table 3), an increase in visit time and an increase in the number of freezing bouts (Figure 3; Table 3). Two other distinct factors for the J strain clearly comprised activity-based measures (distance, rotations, and visits; Figures 3A,D), thus revealing an important distinction between the behavioral structure of conditioned aversion vs. conditioned reward. For the aversion-resistant NJ strain, drug-free (D8-D1 time) and drug-induced NAL-CPA (D9-D1 time) loaded onto two separate factors, Conditioned Aversion and State-Dependent Aversion, each of which contained the same major variables as the J strain (time, freezing bouts, and visit time; Table 4D). Finally, for the NJ strain, a single, third factor comprised change in activity-based measures for Day 8 and Day 9 (Table 4D).

**Table 4:** **Factor analysis of NAL-CPA on the SAL-paired side (left side; the side of retreat)**.

**(A) SAL-trained J mice**					**(B) SAL-trained NJ mice**			
	**Δ Time**	**Δ Freez**.	**D8-D1 Act**.	**D9-D1 Act**.	**Δ Time**	**Δ Freez**.	**D8-D1 Act**.	**D9-D1 Act**.
% variance	19.6	17.4	20.7	21.7	17.9	17.2	20.9	21.5
D8-D1 time	0.82				0.54			
D8-D1 visit time			−0.68				−0.79	
D8-D1 visits			0.96				0.77	
D8-D1 rot.			0.21				0.56	
D8-D1 dist.			0.76				0.84	
D8-D1 freez.		0.96				0.97		
D9-D1 time	0.94				0.74			
D9-D1 visit time	0.75				0.88			
D9-D1 visits				0.75				0.61
D9-D1 rot.				0.86				0.77
D9-D1 dist.				0.98				0.95
D9-D1 freez.		0.97				0.88		
**(C) NAL-trained J mice**					**(D) NAL-trained NJ mice**			
	**Avoid and Freez**.	**D8-D1 Act**.	**D9-D1 Act**.		**Cond. Avoid and Freez**.	**State-Dep. Avoid and Freez**.	**Δ Act**.	
% variance	32.8	19.3	19.5		25.2	25.6	30.7	
D8-D1 time	0.73				0.77			
D8-D1 visit time	0.73				0.80			
D8-D1 visits		0.64			−0.61			
D8-D1 rot.		0.76					0.76	
D8-D1 dist.		0.92					0.83	
D8-D1 freez.	0.72				0.90			
D9-D1 time	0.85					0.80		
D9-D1 visit time	0.82					0.92		
D9-D1 visits			0.74				0.63	
D9-D1 rot.			0.68				0.76	
D9-D1 dist.			0.90				0.90	
D9-D1 freez.	0.86					0.81		

*Freez., freezing bouts; Act., activity, Cond., conditioned; Dep., dependent; Beh., behavior; Pref., preference; dist., distance; rot., rotations*.

To summarize, we identified enhanced NAL-CPA and other concomitant behaviors in J vs. NJ mice as well as a unique behavioral architecture that defined opioid reward vs. opioid aversion. The expression of opioid reward during the drug-free state may be described as one that co-varies with conditioned opioid-like responses in the drug-paired context. In contrast, state-dependent opioid reward does not co-vary with opioid-induced locomotor activity but rather, co-varies with increased visit time. Opioid aversion can best be characterized by longer periods of conditioned and state-dependent avoidance of the NAL-paired side coupled with a marked increase in the number of freezing bouts that is foreshadowed during drug training and is co-expressed with the enhanced avoidance behaviors that underlie NAL-CPA. These results do not modify the interpretation of the main finding (enhanced NAL-CPA in J mice) nor do they suggest that any specific concomitant behavior could replace the measurement of change in time as the primary measure reward or aversion; however, they do provide new insight into the development and expression of the behavioral structure of conditioned opioid reward and aversion and highlight additional variables (e.g., freezing bouts) and relationships that can be examined in future CPP and CPA studies.

This study is limited by the number of doses and types of drugs that could be examined. Future studies examining the behavioral structure of CPP and CPA induced by other pharmacological agents, including psychostimulants, ethanol, and antipsychotics could reveal important distinctions between different drug classes and inform the underlying psychological nature of drug seeking vs. drug avoidance behavior. Furthermore, the potential for genetic differences underlying the behavioral structure of CPP and CPA is an important consideration in forward genetic and reverse genetic engineering experiments and could enrich the interpretation of the data. Finally, because we observed robust B6 substrain differences in NAL-CPA and associated behaviors in the present study, we are currently collecting genotypes and phenotypes from an F_2_ cross derived from these nearly identical substrains to facilitate the identification of QTLs and novel genetic factors that contribute to NAL-CPA (Bryant et al., 2008); this cross has recently been used to identify novel genetic factors contributing to cocaine-induced behaviors (Kumar et al., 2013).

### Conflict of interest statement

The authors declare that the research was conducted in the absence of any commercial or financial relationships that could be construed as a potential conflict of interest.

## Abbreviations

SAL: saline
OXY: oxycodone
NAL: naloxone
CPP: conditioned place preference
CPA: conditioned place aversion
QTL: quantitative trait locus
D4-D2: Day 4—Day 2
D8-D1: Day 8—Day 1
D9-D1: Day 9—Day 1
B6: C57BL/6
J: C57BL/6J
NJ: C57BL/6NJ.
