# Supplementary material for: Behavioral architecture of opioid reward and aversion in C57BL/6 substrains
Source: Front Behav Neurosci. 2015 Jan 12;8:450. doi: 10.3389/fnbeh.2014.00450 (PMC4290583; doi:10.3389/fnbeh.2014.00450)
Supplement: Supplementary file 1 [file Table1.DOCX]

**Supplementary Table 1.** **Change in behavior on the NAL-paired side (right side) following SAL or NAL training**. The mean ± S.E.M. and sample size (N; F = females; M = males.) for each strain (J, NJ) and treatment (SAL, NAL) is presented for each of the 12 variables used in factor analysis.

| **SAL training** | | |
| --- | --- | --- |
| Variable | J (S.E.M.)  N= 56  (30 F, 26 M) | NJ (S.E.M.)  N= 45  (25 F, 20 M) |
| D8-D1 time (s) | 17.6 (20.1) | 12.1 (23.0) |
| D8-D1 visit time (s) | 1.1 (1.1) | 3.5 (3.3) |
| D8-D1 visits | -4.7 (3.5) | -0.3 (2.9) |
| D8-D1 rotations | -7.2 (1.3) | -4.2 (1.6) |
| D8-D1 distance (m) | -3.9 (1.1) | -2.8 (1.1) |
| D8-D1 freezing bouts | 9.8 (16.4) | -8.5 (17.0) |
| D9-D1 time (s) | -0.7 (28.0) | -3.6 (29.9) |
| D9-D1 visit time (s) | 2.1 (1.0) | 1.9 (1.0) |
| D9-D1 visits | -11.4 (4.0) | -5.9 (2.6) |
| D9-D1 rotations | -6.8 (2.3) | -6.6 (1.7) |
| D9-D1 distance (m) | -6.2 (1.8) | -5.6 (1.1) |
| D9-D1 freezing bouts | 7.2 (17.3) | 6.4 (15.1) |
| **NAL training** | | |
| Variable | J (S.E.M.)  N = 44  (22 F, 22 M) | NJ (S.E.M.)  N= 43  (23 F, 20 M) |
| D8-D1 time (s) | -225.5 (26.5) | -73.7 (28.6) |
| D8-D1 visit time (s) | 0.4 (0.5) | -0.5 (0.8) |
| D8-D1 visits | -22.6 (2.9) | -2.3 (2.6) |
| D8-D1 rotations | -14.5 (1.3) | -9.8 (1.9) |
| D8-D1 distance (m) | -12.6 (0.9) | -5.8 (1.2) |
| D8-D1 freezing bouts | 8.1 (8.4) | 29.5 (8.6) |
| D9-D1 time (s) | -299.8 (42.4) | -174.1 (42.9) |
| D9-D1 visit time (s) | 13.2 (9.7) | 0.8 (1.2) |
| D9-D1 visits | -42.8 (3.1) | -13.4 (2.8) |
| D9-D1 rotations | -21.1 (1.1) | -15.0 (1.8) |
| D9-D1 distance (m) | -21.0 (1.0) | -12.6 (1.2) |
| D9-D1 freezing bouts | 26.8 (19.2) | 26.3 (10.4) |
