# Supplementary material for: Behavioral architecture of opioid reward and aversion in C57BL/6 substrains
Source: Front Behav Neurosci. 2015 Jan 12;8:450. doi: 10.3389/fnbeh.2014.00450 (PMC4290583; doi:10.3389/fnbeh.2014.00450)
Supplement: Supplementary file 2 [file Table2.DOCX]

**Supplementary Table 2. Factor analysis of NAL-CPA on the NAL (right)-paired side. (A, B):** Factor analysis of behaviors in NAL-trained mice on the right side. Freez. = freezing bouts; Act. = activity, Cond. = conditioned; Dep. = dependent; Beh. = behavior; Pref. = preference; dist. = distance; rot. = rotations.

| 1. **NAL-trained J mice** | | | | |  | 1. **NAL-trained B6NJ mice** | | |
| --- | --- | --- | --- | --- | --- | --- | --- | --- |
|  | Cond. Avoid. & Freez. | State-Dep. Avoid. & Freez. | D8-D1 Act. | D9-D1 Act. |  | Cond.  Avoid. & Freez. | State-Dep. Avoid. & Freez. | Δ Act. |
| % variance | **19.4** | **21.3** | **17.2** | **18.6** |  | **22.0** | **24.5** | **32.6** |
| D8-D1 time | 0.69 |  |  |  |  |  |  | 0.63 |
| D8-D1 visit time | 0.85 |  |  |  |  | 0.90 |  |  |
| D8-D1 visits |  |  |  | 0.56 |  |  |  | 0.77 |
| D8-D1 rot. |  |  | 0.61 |  |  |  |  | 0.74 |
| D8-D1 dist. |  |  | 0.87 |  |  |  |  | 0.94 |
| D8-D1 freez. | 0.79 |  |  |  |  | 0.80 |  |  |
| D9-D1 time |  | 0.84 |  |  |  |  | 0.94 |  |
| D9-D1 visit time |  | 0.86 |  |  |  |  | 0.68 |  |
| D9-D1 visits |  |  |  | 0.88 |  |  |  | 0.64 |
| D9-D1 rot. |  |  |  |  |  |  | 0.52 |  |
| D9-D1 dist. |  |  |  | 0.81 |  |  |  | 0.68 |
| D9-D1 freez. |  | 0.93 |  |  |  |  | 0.90 |  |
